# Midlife interventions are critical in prevention, delay, or improvement of Alzheimer’s disease and vascular cognitive impairment and dementia

**DOI:** 10.12688/f1000research.11140.1

**Published:** 2017-04-03

**Authors:** Sam Gandy, Tamas Bartfai, Graham V. Lees, Mary Sano

**Affiliations:** 1 Department of Neurology and NFL Neurological Center, Icahn School of Medicine at Mount Sinai, New York, NY, 10029, USA; 2 Department of Psychiatry and Alzheimer’s Disease Research Center, Icahn School of Medicine at Mount Sinai, New York, NY, 10029, USA; 3 Department of Neurochemistry, Stockholm University, Stockholm, 114 18, Sweden; 4 Corpus Alienum Oy, Kirkkonummi, 02400, Finland

**Keywords:** dementia, cognition, cognitive decline, cognitive impairment, amyloid, tau

## Abstract

The basic strategy for focusing exclusively on genetically identified targets for intervening in late life dementias was formulated 30 years ago.  Three decades and billions of dollars later, all efforts at disease-modifying interventions have failed.  Over that same period, evidence has accrued pointing to dementias as late-life clinical phenotypes that begin as midlife pathologies.  Effective prevention therefore may need to begin in midlife, in order to succeed. No current interventions are sufficiently safe to justify their use in midlife dementia prevention trials.  Observational studies could be informative in testing the proposal that amyloid imaging and *APOE*ε *4* genotype can predict those who are highly likely to develop Alzheimer’s disease and in whom higher risk interventions might be justifiable. A naturally occurring, diet-responsive cognitive decline syndrome occurs in canines that closely resembles human Alzheimer’s.  Canine cognitive dysfunction could be useful in estimating how early intervention must begin in order to succeed.  This model may also help identify and assess novel targets and strategies.  New approaches to dementia prevention are urgently required, since none of the world’s economies can sustain the costs of caring for this epidemic of brain failure that is devastating half of the over 85-year-olds globally.

## We are not winning the fight against Alzheimer’s disease

The first generation of the “amyloidocentric” approach to Alzheimer’s has recently drawn to a close, and we are left with the same approved symptomatic treatments that we have had for the past 30 years (i.e., cholinesterase inhibitors). The Alzheimer’s disease-modifying drug discovery field remains at a perfect 100% failure rate when it comes to new approvable disease-modifying interventions. The two most promising amyloid-reducing interventions of 2016, solanezumab ^1^ and verubacestat ^2^, recently failed to modify decline in mild Alzheimer’s disease (AD) and were abandoned.

This is a very serious situation for society, as the disease burden of Alzheimer’s continues to skyrocket globally, yet the private profit-based pharmaceutical companies cannot, and will not, continue working on disease modifying drugs for Alzheimer’s unless convincing new scientific avenues are opened. What this means is that new drug targets must be identified, but for this to happen, we must be open-minded about the trap laid by the early success of identifying genes and molecular mechanisms of the familial Alzheimer’s patients. These early targets presented challenges in druggability, but for many years, these targets seemed scientifically rock solid. Now, this certainty and billions of dollars in trial funding are gone, and many large, prestigious pharmaceutical companies have closed their Alzheimer’s drug discovery programs.

It is time to admit that we are experiencing a rare phenomenon in drug development where the molecular mechanisms uncovered in familial cases of the disease have not helped us manage the sporadic form of the disease. In order to explain our repeated failures, we first blamed the quality of the drug candidates. More recently, we put forward a “kinetic argument”, causing trials to begin earlier and earlier in the course of the disease whilst making little or no effort to identify alternative or additional pathophysiologies beyond amyloidosis and tauopathy. The “kinetic argument” permitted us to remain focused on genetically-predicted drug targets as the most important drug targets, bringing to a halt virtually all other research. As those genetically derived targets continue to fail, experts are commonly overheard to say, “Of course that drug failed: the trial started too late in the progression of the disease; no one expected that to work.” This is revisionist history; it was only five years ago when scientists at several major pharma houses were convinced that the odds of success were high enough to invest $50–100 million for phase 1 and 2 clinical trials, or up to $1 billion each for phase 3 trials, even though each will take 4 years and at least 3 separate successful iterations are required. What was truly surprising to the pharmaceutical industry was that immunotherapies and enzyme inhibitors converging on the same target through entirely independent mechanisms yielded failure after failure, with no new insights. The situation was so surprising that most experts hid their disappointment. As recently as last year, clinicians were telling patients and families how these "anti-amyloid" antibodies and BACE inhibitors were “the most promising drugs ever to enter the pipeline”. To now turn around and say, “no one ever expected these drugs to work” is both disingenuous and hurtful to patients, who respond saying, “If no one expected that drug to work, then why did you recommend the trial to me?”

Each successively earlier trial failure condemns doctors and patients to another 5 to 7 years of purgatory while the next trial iteration moves five clicks earlier in enrollment age. Yet these trial designs move forward without sufficient ability to define the molecular pathology with precision at the level of the individual patient. This argues not only for diversifying the disease-modifying portfolio but also for redoubling efforts on symptomatic interventions that are also easier to get through the regulatory process.

## Can we pinpoint how early is “early enough”?

Perhaps we should seek specific, empirical data about how early is “early enough” in humans. Patients with epilepsy and *APOEε4* alleles do not develop Alzheimer’s yet they deposit plaques in their early 40s ^3^. This rare but surprisingly early phenomenon argues strongly that we should initiate intervention to prevent amyloidosis or tauopathy not just a little earlier, as we are doing now, but far earlier; in other words, we should intervene in midlife, not later in life. This dovetails well with evidence that midlife risk factors lead to a late-life phenotypes. Midlife-onset hypertension is a risk factor for AD in late life; late life-onset hypertension appears to be *protective* of cognition ^4^. What sorts of interventions might these be? Dietary interventions with small effect size but employed over decades might have important cumulative effects. Vaccines may provide lifelong or very long interventions when the proper antigens and adjuvants are identified and employed.

What might we do to refine our guess at what might be truly “early enough” for us to intervene? One might design an observational study of *APOEε4* carriers in their 40s ^3, 5^. Annual amyloid and tau imaging of *APOEε4* carriers could be used to identify those with evidence, or at highest risk, of progression ^5^. Once an *APOEε4* carrier becomes amyloid-imaging positive, one could imagine entering them either into an observational study or into an authentic trial employing reducers of amyloid, tau, or both. An advantage of this design is the possibility that for serially imaged subjects observed to change from negative to positive proteinopathy imaging, it will be possible to know that the proteinopathy has been present for 12 months or less. If we jump back in age as far as the mid 40s, and show that we can engage the proteinopathy targets at that early point, but yet *still* fail clinically, that will be a strong indication that “anti-proteinopathy-only” will never succeed.

## Mixed pathology may be the most common underpinning for dementia

It is worth keeping in mind that even if we have impact on Alzheimer’s pathology, the frequent concurrent presence of multiple pathologies will continue to confound. These days, there is more research on the relationship between vascular cognitive impairment and dementia (VCID) and Alzheimer’s dementia, but not yet enough, even though defining this is probably profoundly important. Among African Americans with clinical Alzheimer’s, 70% have mixed pathology at autopsy ^6^. Despite much attention being given to insulin signaling and brain proteinopathy, virtually all the accumulated data indicate that dementia of type-2 diabetes is not Alzheimer’s but is primarily vascular in origin ^7^. Synucleinopathy, which is present in about 1/3 of Alzheimer’s patients, induces a plethora of epigenetic changes in the brain transcriptome ^8^. This means that, currently, perfect antemortem diagnosis is sometimes impossible. Certainly, the more mixed dementia subjects there are in a trial intended to assess the efficacy of a drug that only treats Alzheimer’s, the lower the sensitivity to see a signal will be. There is a good chance that each of the various underlying pathogenetic mechanisms will have their own optimum time window for intervention, and that this will have to be factored in as well. On the other hand, improved midlife cardiovascular health could benefit cognition and may delay *all* causes of dementia.

## Immunology of cognitive decline in Alzheimer’s disease

While genetics and, more recently, multi-scale network-level genomics ^9^ have taught us, and continue to teach us, much about the molecular pathology of Alzheimer’s, imaging and pathology have taught us just as much about the disappointingly poor clinicopathological and clinicoradiological correlation in this illness. Cognitive decline is poorly predicted by amyloid imaging in isolation, as was foretold by the Religious Orders study some 20 years ago ^10^.

Are there truly new drug targets? What might we be missing in our formulation of the pathogenesis of sporadic Alzheimer’s? Of the two dozen genes linked by genome-wide association studies (GWAS), two thirds are lipid- or immune-related ^11^, raising the possibility of a neuroinflammatory/neurodegenerative dementia-causing pathway that might be worthy of further research. DeStrooper has recently proposed an alternative formulation of AD as a clinical umbrella under which “feed-forward” pathogenesis scenarios lie (e.g., inflammation causes or exacerbates tauopathy; then, in turn, tauopathy aggravates inflammation) ^12^. This model fits the existing data at least as well as the classical linear amyloid hypothesis and explains some of the holes in the current predictive models. DeStrooper’s formulation ^12^ includes scenarios where anti-proteinopathy drugs alone would probably be inadequate. Along the same lines, the *CR1* risk polymorphism is associated with increased risk for clinical Alzheimer’s but in the setting of progression-related reduction in amyloidosis ^13, 14^.

One other model might be worth investigating: Canine cognitive dysfunction (CCD) ^15^ is the only naturally occurring mammalian dementia to mimic Alzheimer’s disease. In CCD, diet and lifestyle have measurable impact on disease progression ^15^. The CCD model could contribute to our understanding of how early intervention must begin in order to be effective. And with CCD, one could test drugs as prophylaxis *in vivo*. In the meantime, the importance and potential benefit of safe and easy diet and lifestyle changes for humans should be a topic of much stronger advocacy. In fact, strong evidence that better cardiovascular health reduces prevalence of both Alzheimer’s and VCID is already beginning to emerge and must be a clear part of patient education by clinicians ^16– 23^. To its credit, the April 2017 issue of *Scientific American* heralds “Success in the Fight Against Alzheimer’s”, a review of dementia risk reduction benefit that can be realized through modification of diet and lifestyle beginning in midlife ^24^.

## There may be more “unknown unknowns” yet to be revealed

Finally, it is important to emphasize that researchers tackling Alzheimer’s and other dementias must remain clear-eyed, challenged, and worried that there are still many gaps in our understanding. A simple backward jump to earlier intervention may require 5 more iterations at the current pace, if indeed we should be intervening when subjects are in their 40s. If the disease can be primarily driven by lipid pathology or inflammatory pathology even with little or no protein aggregate pathology as could be inferred from the nature of the GWAS hits ^11^, and from the variability in amyloid-first phenotypic sequences vs. neurodegeneration-first phenotypic sequences ^25^, then efforts focused solely on purging clumped proteins from brains in which they may have lingered silently for decades may always come up short.
