# Supplementary material for: Midlife interventions are critical in prevention, delay, or improvement of Alzheimer’s disease and vascular cognitive impairment and dementia
Source: F1000Res. 2017 Apr 3;6:413. [Version 1] doi: 10.12688/f1000research.11140.1 (PMC5399952; doi:10.12688/f1000research.11140.1)
Supplement: Peer review history

# Peer review: Referee response for version 1

**DOI:** 10.5256/f1000research.12018.r21956

**Published:** 2017-04-19

**Authors:** Domenico Pratico

**Affiliations:** 1 Department of Pharmacology, Center for Translational Medicine, Lewis Katz School of Medicine, Temple University, Philadelphia, PA, USA

This is a very interesting and stimulating opinion article in which the authors try to make sense of the current state of the Alzheimer’s disease (AD) pharmacotherapy.

 They present in a very objective fashion the disappointing scenario we are facing in this area, and at the same time they provide very insightful food for thought on how the research should move forward.

 I would like the authors to spend some additional words on the followings:

 1.            The multifactorial nature of sporadic AD, and because of that a multi-target and may be personalized approach versus a one-size-fits-all solution is more likely to work.

 2.            While the large GWAS studies have provided evidence for good genetic leads, there is strong evidence indicating that environmental factors (i.e., lifestyle, diet) can ultimately influence the clinical phenotype.

I have read this submission. I believe that I have an appropriate level of expertise to confirm that it is of an acceptable scientific standard.

# Peer review: Referee response for version 1

**DOI:** 10.5256/f1000research.12018.r21939

**Published:** 2017-04-18

**Authors:** W.Sue T Griffin, Orwa Aboud

**Affiliations:** 1 Department of Geriatrics, University of Arkansas for Medical Sciences, Little Rock, AR, USA

Dr. Gandy and his colleagues have come to the heart of the matter – current anti-amyloid strategies have failed to change the course of Alzheimer’s disease (AD). This important article is written as an “Aha, we can now address the question of what other strategies might hold promise toward preventing or delaying onset of Alzheimer’s disease (AD).” They raise a number of very important questions, including how early should we treat; what is the role of multiple pathologies in Alzheimer pathogenesis; are the pathways that give rise to the neuronal dysfunction and loss noted in AD the same ones that obtain in the pathogenesis of other neurodegenerative diseases; are such pathways known and, if so, have the drivers of these pathways been identified? Most of these questions have been addressed in animal models and some have included evidence of the existence of such pathways in brain tissue from patients vs controls. A great deal of effort has been given to one of the areas of interest noted by the authors “Immunology of cognitive decline in AD.” The authors’ discussion of a recent review of the potential relationship between lifestyle changes such as diet and exercise and how this may have a beneficial effect as a middle-age combatant of cognitive decline is laudable. However, implementation of such lifestyle changing practices would require individuals to be either coerced or otherwise convinced to adhere to such practices. At present, even though there is convincing evidence of tangible benefits of exercise and diet ^1^, the data is not with us regarding adherence to such regimens. Therefore, we would like to suggest inflammation, both in the brain and in the periphery, as a prime target for early intervention in Alzheimer pathogenesis through the use of currently available drugs or drugs developed so as to have fewer or more tolerable side effects. Data from the Framingham study of 691 cognitively intact individuals provide evidence of spontaneous increases in production by blood monocytes of two pluripotent proinflammatory cytokines, interleukin-1 (IL-1) and TNFα ^2^; these increases were suggested to serve as markers that “strengthen” a link between inflammation and development of AD. In addition, an accumulation of epidemiological data has consistently sided with use of anti-inflammatory compounds as increasing the odds against being in the “Alzheimer group” and in favor of being in the non-demented, non-neuropathologically confirmed group. Data from the Rotterdam study of non-NSAID users vs NSAID users reported an adjusted relative risk ratio of 0.54 among NSAID users as support for the potential of NSAIDs to protect against development of AD ^3^. In a larger study of the VA database (~50,000 clinically and neuropathologically confirmed AD patients vs ~200,000 non-AD patients), 5 or more years of ibuprofen use reduced the adjusted odds ratio to 0.56 ^4^. Further in the extended results of the ADAPT study, asymptomatic subjects who received naproxen had a reduced incidence of AD compared to those who received either celecoxib or placebo ^5^.

 In view of the evidence implicating inflammation as increasing risk for later development of AD, it seems prudent to first explore what we know at present regarding early events in Alzheimer pathogenesis as such events may presage development, or help us pinpoint “when is the best time to intervene” to forestall formation of the aggregate defining neuropathological hallmarks of AD. In the McGeers’ early studies of neuroinflammation in AD, immune markers were identified on plaque-associated microglia in AD brain ^6^. The following three discoveries: one showing that activated glia in AD brain overexpress IL-1, a second showing that microglial activation with overexpression of interleukin-1 (IL-1) as well as astrocyte activation and overexpression of the neurite-growth stimulating cytokine S100B are prominent in fetuses, newborns, children, and adults ^7^, and a third showing that IL-1 induces synthesis of APP in human cell cultures ^8^ opened a new field of investigation, viz., the potential of IL-1-directed pathways to promote neuropathogenesis. These two cytokines, IL-1 and S100B upregulate each other and both induce synthesis of βAPP in vitro and in vivo. This βAPP may then be cleaved for β-amyloid (Aβ) plaque formation or for sAPPα for further microglial activation and overexpression and release of IL-1β ^9^^-^^10^. Moreover, IL-1β, via its ability to induce synthesis as well as activation of MAPK-p38, increases production of hyperphosphorylated tau, for formation of the paired helical filaments in neurofibrillary tangles, as well as α-synuclein for formation of Lewy bodies ^11^. If such neuroinflammation, which has now been associated with immune challenges in the periphery is associated with risk for development of AD, it would seem prudent to develop an inflammatory index in order to decide when treatment should begin. In view of the appearance of neuroinflammation, noted as increased activation of glia and overexpression of IL-1 decades before frank pathology in Down’s syndrome, perhaps erring on the side of caution, beginning treatment sooner rather than later may be preferable.

We have read this submission. We believe that we have an appropriate level of expertise to confirm that it is of an acceptable scientific standard.

## References

[1] ImayamaIUlrichCMAlfanoCMWangCXiaoLWenerMHCampbellKLDugganCFoster-SchubertKEKongAMasonCEWangCYBlackburnGLBainCEThompsonHJMcTiernanA: Effects of a caloric restriction weight loss diet and exercise on inflammatory biomarkers in overweight/obese postmenopausal women: a randomized controlled trial. *Cancer Res*.2012;72(9) : 10.1158/0008-5472.CAN-11-3092 2314-26 10.1158/0008-5472.CAN-11-3092 22549948PMC3342840

[2] TanZSBeiserASVasanRSRoubenoffRDinarelloCAHarrisTBBenjaminEJAuRKielDPWolfPASeshadriS: Inflammatory markers and the risk of Alzheimer disease: the Framingham Study. *Neurology*.2007;68(22) : 10.1212/01.wnl.0000263217.36439.da 1902-8 10.1212/01.wnl.0000263217.36439.da 17536046

[3] AndersenKLaunerLJOttAHoesAWBretelerMMHofmanA: Do nonsteroidal anti-inflammatory drugs decrease the risk for Alzheimer's disease? The Rotterdam Study. *Neurology*.1995;45(8) :1441-5 764403710.1212/wnl.45.8.1441

[4] VladSCMillerDRKowallNWFelsonDT: Protective effects of NSAIDs on the development of Alzheimer disease. *Neurology*.2008;70(19) : 10.1212/01.wnl.0000311269.57716.63 1672-7 10.1212/01.wnl.0000311269.57716.63 18458226PMC2758242

[5] BreitnerJCBakerLDMontineTJMeinertCLLyketsosCGAsheKHBrandtJCraftSEvansDEGreenRCIsmailMSMartinBKMullanMJSabbaghMTariotPNADAPT Research Group: Extended results of the Alzheimer's disease anti-inflammatory prevention trial. *Alzheimers Dement*.2011;7(4) : 10.1016/j.jalz.2010.12.014 402-11 10.1016/j.jalz.2010.12.014 21784351PMC3149804

[6] McGeerPLMcGeerEG: History of innate immunity in neurodegenerative disorders. *Front Pharmacol*.2011;2: 10.3389/fphar.2011.00077 77 10.3389/fphar.2011.00077 22144960PMC3228970

[7] GriffinWSStanleyLCLingCWhiteLMacLeodVPerrotLJWhiteCLAraozC: Brain interleukin 1 and S-100 immunoreactivity are elevated in Down syndrome and Alzheimer disease. *Proc Natl Acad Sci U S A*.1989;86(19) :7611-5 252954410.1073/pnas.86.19.7611PMC298116

[8] GoldgaberDGoldfarbLGBrownPAsherDMBrownWTLinSTeenerJWFeinstoneSMRubensteinRKascsakRJ: Mutations in familial Creutzfeldt-Jakob disease and Gerstmann-Sträussler-Scheinker's syndrome. *Exp Neurol*.1989;106(2) :204-6 257245010.1016/0014-4886(89)90095-2

[9] BargerSWHarmonAD: Microglial activation by Alzheimer amyloid precursor protein and modulation by apolipoprotein E. *Nature*.1997;388(6645) : 10.1038/42257 878-81 10.1038/42257 9278049

[10] LiYLiuLBargerSWGriffinWS: Interleukin-1 mediates pathological effects of microglia on tau phosphorylation and on synaptophysin synthesis in cortical neurons through a p38-MAPK pathway. *J Neurosci*.2003;23(5) :1605-11 1262916410.1523/JNEUROSCI.23-05-01605.2003PMC3833596

[11] GriffinWSLiuLLiYMrakREBargerSW: Interleukin-1 mediates Alzheimer and Lewy body pathologies. *J Neuroinflammation*.2006;3: 10.1186/1742-2094-3-5 5 10.1186/1742-2094-3-5 16542445PMC1435743
